# Personality traits: an important factor affecting fear of movement in dialysis patients

**DOI:** 10.3389/fpsyt.2025.1574232

**Published:** 2025-07-30

**Authors:** You Zhang, Ruike Zhang, Xiaoyong Miao, Maoting Li, Lingyan Zhang, Lu Li, Yuting Liu, Qing Shao, Lingling Ding, Tong Su, Zhiyong Guo, Nanmei Liu

**Affiliations:** ^1^ Department of Nephrology, Changhai Hospital, Naval Medical University, Shanghai, China; ^2^ Faculty of Psychology, Naval Medical University, Shanghai, China; ^3^ Department of Nephrology, Naval Medical Center of PLA, Naval Medical University, Shanghai, China

**Keywords:** personality, extraversion, emotional stability, conscientiousness, openness to experience, dialysis

## Abstract

**Background:**

This study investigated predictive factors and personality determinants of kinesiophobia (fear of movement) in dialysis patients. Methods: Using the Chinese versions of the Ten-Item Personality Inventory (TIPI-C) and Tampa Scale for Kinesiophobia Heart (TSK-SV heart), we assessed 329 patients from December 2022 to February 2023. The influencing factors of fear of movement were identified by univariate analysis and multiple linear regression.

**Results:**

The mean score of fear of movement for dialysis patients was 45.128 (45.128 ± 7.023). Multiple linear regression revealed that advanced age,local medical insurance, and lower education significantly predicted higher kinesiophobia scores, while exercise habits, being married, alcohol and tobacco use served as protective factors. Personality analysis demonstrated that lower extraversion, emotional stability, and conscientiousness were associated with greater kinesiophobia.

**Conclusions:**

Personality determinants were significantly associated with fear of movement. These findings emphasize the clinical utility of personality assessment in identifying high-risk patients and personalizing rehabilitation strategies for this vulnerable population.

## 1 Introduction

Physical activity constitutes a cornerstone of chronic disease management (1–4), with particular demonstrated efficacy across the continuum of renal disease (3, 5–9). For end-stage kidney disease (ESKD) patients undergoing dialysis, physical dysfunction represents a significant contributor to adverse clinical outcomes (10). Robust evidence confirms that structured exercise interventions yield clinically meaningful improvements in both physical capacity and health-related quality of life (HRQoL) in chronic kidney disease (CKD) patients (11–15), with multicenter studies (n=171 hemodialysis patients) documenting sustained benefits from resistance training (16) and randomized controlled trials validating comparable advantages from home-based programs in both hemodialysis and peritoneal dialysis populations (11). These therapeutic effects are mediated through measurable physiological adaptations including enhanced muscular strength (17) and improved blood pressure control (18, 19).

However, the implementation of exercise therapy faces a formidable psychological barrier in kinesiophobia - a pathological fear of movement (20) that perpetuates physical inactivity cycles (21). This phenomenon proves particularly pronounced in kidney transplant recipients(KTRs) exhibiting excessive graft-protective behaviors (22, 23). Contemporary psychological research reveals that temperament and personality traits fundamentally modulate behavioral responses to physical activity (24). Patients with kinesiophobia typically demonstrate pessimistic outcome expectations and harm-avoidant personality profiles (25), often displaying impaired self-assessment capabilities where exercise tolerance evaluations are disproportionately influenced by prior negative experiences rather than objective capacity (25–27). Emerging research further supports personality-mediated biases in self-reported exercise assessments (28).

Cultural health beliefs prioritizing rest over physical activity compound these psychological dimensions (29, 30). This cognitive-cultural confluence frequently manifests as exercise hypervigilance and subsequent avoidance (31–33), establishing kinesiophobia as a personality-anchored behavioral response specific to physical activity contexts (34). These observations align with broader clinical evidence that psychological factors - particularly depression, anxiety, and kinesiophobia - disproportionately influence rehabilitation outcomes compared to biological or social determinants (35–37), carrying significant implications for treatment adherence and quality of life (38–40).

The diathesis-stress model provides a theoretical framework for these phenomena, positing that pre-existing personality traits amplify during health stressors, predisposing individuals to maladaptive responses (41). Neuroticism exemplifies this mechanism through its established associations with: (1) catastrophic pain cognition, (2) subsequent kinesiophobia development, and (3) ultimately, impaired pain adaptation and depressive symptomatology (42, 43).

Given (1) the established efficacy of exercise therapy in dialysis populations (3), (2) the significant association between kinesiophobia and reduced physical activity (44, 45), and (3)growing recognition of personality’s role in health behavior modulation, this study systematically investigates personality determinants of kinesiophobia in dialysis patients. Addressing this knowledge gap represents a critical step in optimizing rehabilitation outcomes for this vulnerable population through targeted intervention strategies.

## 2 Materials and methods

### 2.1 Study design and participants

A prospective cohort study was conducted following *a priori* power analysis using G*Power 3.1.Based on one-way ANOVA calculations (effect size f = 0.25, α= 0.05, power = 0.8, 4 groups), the minimum required sample size was determined to be 180 participants. Additional analyses were performed for independent t-tests, Pearson correlation, and multiple linear regression to ensure comprehensive power assessment. We recruited 329 end-stage kidney disease patients undergoing maintenance dialysis at Changhai Hospital and Naval Medical Center of PLA between December 2022 and February 2023. Inclusion criteria comprised: (1) age≥18 years, (2) Chinese language fluency, and (3) current dialysis treatment. Exclusion criteria included severe cognitive impairment or diagnosed mental illness. After excluding incomplete responses, 329 participants (179 male, 150 female) were included in final analyses, yielding a 99.1% response rate.

The study was approved by the Research Ethics Commission of Naval Medical Center of PLA (protocol code 2022121903).

### 2.2 Measures

We collected comprehensive demographic data including age, gender, educational attainment, marital status, cohabitation with children, medical insurance coverage, and pre-diagnosis health behaviors (tobacco/alcohol use and exercise frequency). Clinical variables encompassed primary renal diagnosis, initial and current dialysis modalities, and treatment duration.

Personality characteristics were assessed using the Chinese Version of the Ten-Item Personality Inventory (TIPI-C), a validated adaptation of the original TIPI for Chinese populations. This 10-item instrument evaluates five core personality dimensions:Extraversion (Characterized by enthusiasm, positive emotionality, and sociability), Agreeableness(Reflecting compassion, cooperativeness, and trust), Conscientiousness(Indicating self-discipline, determination, and preference for planned behavior), Emotional Stability(Representing confidence and low emotional reactivity) and Openness(Encompassing creativity, imagination, and novelty-seeking). The TIPI-C has demonstrated satisfactory reliability and validity in Chinese cultural contexts (38, 39).

Emotional stability reflects psychological resilience, characterized by confidence, security, and reduced emotional reactivity in stressful situations. Its counterpart, neuroticism, indicates heightened vulnerability to emotional instability. Openness to experience captures cognitive flexibility and novelty-seeking tendencies, manifesting as creativity, inventiveness, and receptivity to unconventional ideas. Individuals with low openness typically demonstrate conventional attitudes and resistance to change. These personality dimensions were assessed using a standardized 7-point Likert scale (1 = strongly disagree to 7 = strongly agree). The Chinese version of the Ten-Item Personality Inventory (TIPI-C) was adapted from the original TIPI to assess personality traits in Chinese populations. Psychometric evaluations have confirmed its reliability and validity for this cultural context (46, 47).

For our primary outcome, kinesiophobia (fear of movement), we administered the validated Chinese version of the Tampa Scale for Kinesiophobia Heart (TSK-SV Heart). This 17-item instrument measures four domains:Danger (perception of disease risk), Fear (fear of adverse outcomes of exercise), Avoidance (avoidance of exercise due to heart problems), and Dysfunction (physical, psychological, and social dysfunction caused by fear of movement). All items employ a 4-point Likert scale (1 = strongly disagree to 4 = strongly agree), with higher scores indicating greater kinesiophobia severity (48).

### 2.3 Statistical analysis

All statistical analyses were performed with the use of SPSS 26.0 (Statistical Package for the Social Sciences) for Windows (SPSS, Chicago, IL).A two-sided P<0.05 was considered statistically significant.

For univariate analysis, statistical differences were analyzed using Student’s t-test or one-way ANOVA test. Pearson correlation analysis was used to analyze correlations between continuous variables.

Univariate analysis was conducted for each variable, and multivariate analysis was used for factors with P value<0.05 in univariate analysis. Multiple linear regression analysis using the stepwise procedure was conducted for detecting risk factors for danger, fear, avoidance, dysfunction and fear of movement total score.

## 3 Results

### 3.1 Baseline information

The study cohort comprised 329 dialysis-dependent patients with kidney disease (mean age = 58.599years; range: 22–97 years). Demographic characteristics revealed that 83% were married, 36.8% cohabitated with children, and 92.4% possessed local medical insurance. Educational attainment distribution included: 12.8% with bachelor’s degrees or higher, 45.0% with high school/junior college education, and 42.2% with middle school education or less. Current tobacco or/and alcohol use was reported by 28 participants(8.5%). Primary renal diagnoses were: diabetic nephropathy (20.1%), hypertensive nephropathy (19.8%), chronic nephritis (24.0%), and other etiologies (36.2%). Initial dialysis modalities included hemodialysis (70.8%) and peritoneal dialysis (29.2%), with current treatment distribution of 78.4% hemodialysis and 21.6% peritoneal dialysis/hemodialysis +peritoneal dialysis.The five personality traits of the patients are emotional stability (9.447 ± 1.865), extraversion (8.684 ± 3.130), openness (8.672 ± 2.879), agreeableness (10.185 ± 1.691), and conscientiousness (9.462 ± 2.085) (**Table 1**).

**Table 1:** Sociodemographic variables of the subjects.

Characteristics	N or Mean	Frequency or SD
Gender		
Male	179	54.4%
Female	150	45.6%
Age	58.599	14.836
Marital status		
Married	273	83%
Other	56	17%
Live with child		
yes	121	36.8%
no	208	63.2%
Medical insurance		
Local	304	92.4%
Not local	25	7.6%
Education		
Junior middle school or below	139	42.2%
High school or junior college	148	45.0%
Bachelor degree or above	42	12.8%
Alcohol and tobacco use		
yes	28	8.5%
no	301	91.5%
Exercise		
yes	149	45.3%
no	180	54.7%
Primary Disease		
Diabetic Nephropathy	66	20.1%
Hypertensive renal damage	65	19.8%
Chronic Nephritis	79	24.0%
Other	119	36.2%
Initial dialysis modality		
Hemodialysis(HD)	233	70.8%
Peritoneal dialysis (PD)	96	29.2%
Current dialysis modality		
HD	258	78.4%
PD/HD+PD	71	21.6%
Dialysis duration	6.684	5.436
Personality variables		
Extraversion	8.684	3.130
Agreeableness	10.185	1.691
Conscientiousness	9.462	2.085
Emotional Stability	9.447	1.865
Openness	8.672	2.879

HD, Hemodialysis; PD, peritoneal dialysis.

The TSK-SV heart scale measured participants’ scores on four fear of movement factors: Danger (9.556 ± 1.246), Fear (12.219 ± 2.184), Avoidance (13.982 ± 3.206), and Dysfunction (9.371 ± 2.211) (**Table 2**).

**Table 2:** Scores of danger, fear, avoidance, dysfunction and fear of movement total scores.

Fear of movement	Mean	SD
Danger	9.556	1.246
Fear	12.219	2.184
Avoidance	13.982	3.206
Dysfunction	9.371	2.211
Fear of movement total scores	45.128	7.023

### 3.2 Danger, fear, avoidance, dysfunction and TSK-SV Heart total scores according to demographic variables and nephropathy variables

Significant differences in kinesiophobia scores emerged across sociodemographic and clinical subgroups (**Table 3**). Compared to married participants, unmarried individuals (single/divorced/widowed) demonstrated elevated danger scores (t=-2.474, p=0.014) but reduced fear scores (t=2.454, p=0.015). Participants with local medical insurance showed significantly greater fear (t=3.334, p=0.001), avoidance (t=3.604, p<0.001), and total kinesiophobia scores (t=2.789, p=0.006) than those without insurance. Age exhibited positive correlations with fear, avoidance, dysfunction, and total scores. Educational attainment significantly influenced fear (F=8.839, p<0.001), avoidance (F=7.565, p=0.001), and fear of movement total scores (F=7.817, p<0.001). Regular exercise was associated with substantially lower scores across all domains: danger (t=-4.869, p<0.001), fear (t=-8.617, p<0.001), avoidance (t=-10.883, p<0.001), dysfunction (t=-9.876, p<0.001), and fear of movement total scores (t=-12.600, p<0.001). Tobacco/alcohol users reported heightened fear (t=-2.660, p=0.008) and avoidance (t=-2.326, p=0.021) compared to non-users. In addition, primary disease etiology showed significant associations with danger and dysfunction scores, while both initial and current dialysis modalities correlated with fear, avoidance, and total kinesiophobia scores (**Table 3**).

**Table 3:** Danger, fear, avoidance, dysfunction and TSK-SV Heart total scores according to demographic variables and nephropathy variables.

Variables	Danger	Fear	Avoidance	Dysfunction	Fear of movement total scores
	Mean±SD	Mean±SD	Mean±SD	Mean±SD	Mean±SD
Demographic variables					
Gender					
Male	9.670±1.203	12.117±2.282	14.134±3.221	9.492±2.224	45.413±7.094
Female	9.420±1.286	12.340±2.062	13.800±3.190	9.227±2.193	44.787±6.945
*F/t*	1.822	-0.921	0.941	1.083	0.806
Marital status					
Married	9.480±1.219	12.352±2.132	14.125±3.189	9.377±2.210	45.333±6.958
Other	9.929±1.319	11.571±2.334	13.286±3.229	9.339±2.234	44.125±7.311
*F/t*	-2.474*	2.454*	1.789	0.117	1.174
Live with child					
yes	9.595±1.364	11.967±2.179	13.744±3.100	9.372±2.134	44.678±6.883
no	9.634±1.175	12.365±2.178	14.120±3.266	9.370±2.259	45.389±7.106
*F/t*	0.430	-1.600	-1.027	0.007	-0.886
Medical insurance					
Local	9.533±1.218	12.332±2.138	14.161±3.192	9.408±2.196	45.434±6.964
Not local	9.840±1.546	10.840±2.304	11.800±2.550	8.920±2.379	41.400±6.795
*F/t*	-1.185	3.334**	3.604***	1.061	2.789**
Education					
Junior middle school or below	9.727±1.262	12.374±2.253	14.345±3.243	9,525±2.178	45.971±7.111
High school or junior college	9.446±1.174	12.439±1.966	14.135±3.021	9.412±2.237	45.432±6.550
Bachelor degree or above	9.381±1.396	10.929±2.289	12.238±3.237	8.714±2.156	41.262±7.245
*F/t*	2.313	8.839***	7.565**	2.234	7.817***
Alcohol and tobacco use					
yes	9.714±1.272	11.179±2.554	12.643±2.896	9.393±2.409	42.929±7.050
no	9.542±1.245	12.316±2.125	14.106±3.210	9.369±2.196	45.332±6.997
*F/t*	0.701	-2.660**	-2.326*	0.055	-1.738
Exercise					
yes	9.201±1.219	11.188±1.879	12.208±2.246	8.208±1.883	40.805±4.809
no	9.850±1.193	13.072±2.050	15.450±3.143	10.333±1.992	48.706±6.544
*F/t*	-4.869***	-8.617***	-10.883***	-9.876***	-12.600***
Age					
*r*	-0.014	0.454**	0.469**	0.306**	0.449**
Nephropathy variables					
Primary disease					
Diabetic Nephropathy	9.667±1.194	12.485±2.040	14.394±3.048	9.561±2.234	46.706±6.751
Hypertensive Renal damage	9.585±1.211	12.000±2.598	14.231±3.512	9.723±2.478	45.539±7.856
Chronic nephritis	9.873±1.067	11.911±2.283	13.899±3.112	9.798±1.814	45.481±6.439
Other	9.269±1.351	12.395±1.923	13.672±3.184	8.790±2.186	44.126±7.027
*F/t*	4.114**	1.328	0.881	4.578**	1.379
Initial dialysis modality					
Hemodialysis(HD)	9.537±1.200	12.489±2.215	14.356±3.243	9.464±2.221	45.846±7.183
Peritoneal Dialysis (PD)	9.604±1.357	11.563±1.967	13.073±2.939	9.146±2.181	43.385±6.320
*F/t*	-0.447	3.561***	3.351**	1.186	3.081**
Current dialysis modality					
HD	9.516±1.226	12.411±2.202	14.314±3.202	9.434±2.173	45.674±7.064
PD/HD+PD	9.704±1.314	11.521±1.977	12.775±2.938	9.141±2.344	43.141±6.541
*F/t*	-1.131	3.079**	3.649***	0.990	2.718**
Dialysis duration					
*r*	-0.043	-0.054	0.016	0.003	-0.016

HD, Hemodialysis; PD, peritoneal dialysis.

*p < 0.05, **p <0.01, ***p < 0.001.

Five personality traits were all significantly associated with danger, avoidance, dysfunction, and fear of movement total score, however fear was significantly associated with conscientiousness and openness (**Table 4**).

**Table 4:** Pearson correlation analysis (r, P-value) for factors associated with fear of movement.

Variables	Danger	Fear	Avoidance	Dysfunction	Fear of movement total scores
	R	R	R	R	R
Extraversion	-0.242***	-0.065	-0.220***	-0.375***	-0.282***
Agreeableness	-0.191***	-0.050	-0.143**	-0.226***	-0.186**
Conscientiousness	-0.237***	-0.220***	-0.305***	-0.345***	-0.358***
Emotional Stability	-0.258***	-0.100	-0.150**	-0.294***	-0.238***
Openness	-0.354***	-0.177**	-0.346***	-0.466***	-0.422***

*p < 0.05, **p <0.01, ***p < 0.001.

### 3.3 Risk factors for fear of movement scores

**Figure 1:**
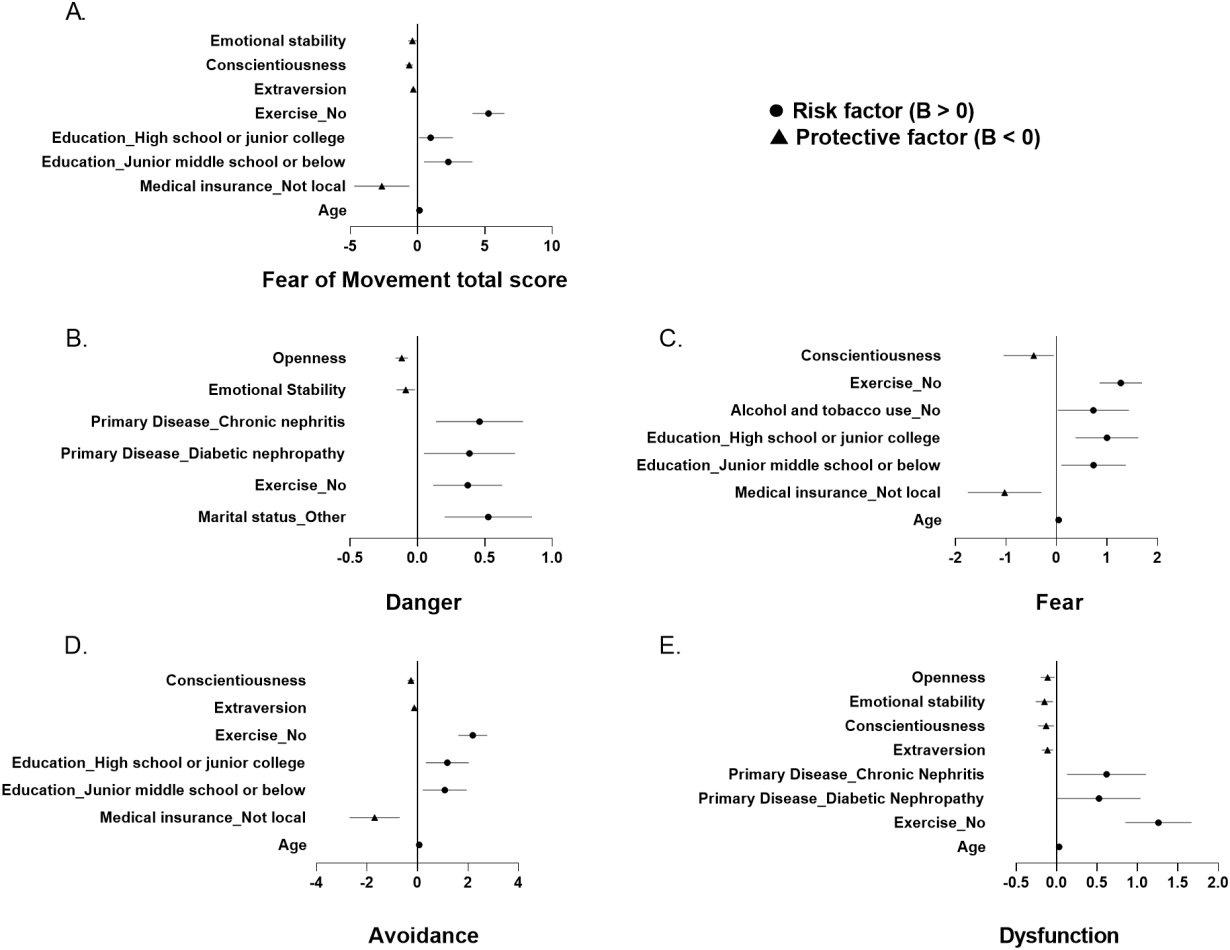
Forest plots of final multivariable linear regression models for **(A)** Fear of Movement total score and **(B-E)** its subscales. Unstandardized coefficients **(B)** with 95% confidence intervals are shown for significant predictors retained in the final model, selected from candidate variables with p<0.05 in univariate analyses. Continuous variables represent the change in outcome per unit increase (e.g., age: 1 year). Categorical variables are relative to reference groups: Education (ref: Bachelor degree or above), Exercise (ref: yes), Marital status (ref: Married), Medical insurance (ref: Local), Alcohol and tobacco use (ref: yes). The vertical line indicates null effect (B=0). Panel descriptions: **(A)** Fear of Movement total score. **(B)** Danger. **(C)** Fear. **(D)** Avoidance. **(E)** Dysfunction. Initial candidate variables not retained in the final model were included in model building but did not reach significance (p≥0.05).

Multiple linear regression analyses were conducted to identify risk factors for kinesiophobia subscales (danger, fear, avoidance, dysfunction) and total scores (**Figure 1**). Univariate analysis revealed significant associations between danger scores and marital status, exercise habits, primary disease diagnosis, and all five personality dimensions.

The final regression model demonstrated that marital status (married), regular exercise, higher emotional stability, and greater openness served as protective factors against danger perceptions. Conversely, diabetic nephropathy and chronic nephritis emerged as significant clinical risk factors (**Figure 1B**; **Supplementary Table S1**).

The fear prediction model demonstrated statistically significant explanatory power (F = 26.508, p < 0.001), which explained 35.2% of the variation in the fear variable (adjusted R^2^ = 0.352). Significant risk factors included advanced age, local medical insurance coverage, lower educational attainment, and physical inactivity. Conversely, conscientiousness emerged as a protective factor (**Figure 1C**; **Supplementary Table S2**).

For avoidance behaviors, the regression model (adjusted R² = 0.442) identified age, local insurance, and education level as risk factors. Protective factors included regular exercise, extraversion, and conscientiousness (**Figure 1D**; **Supplementary Table S3**).

The linear regression had dysfunction as the dependent variable to be explained by several independent variables including age, habits of exercising, primary disease, extraversion, conscientiousness, emotional stability, and openness(adjusted R2 = 0.414). Participants with older age, no habit of exercising, and having diabetic nephropathy or chronic nephritis as a primary disease had higher levels of dysfunction. Those with higher levels of extraversion, conscientiousness, emotional stability, and openness had lower levels of dysfunction (**Figure 1E**; **Supplementary Table S4**).

The predictive model of the fear of movement total score was constructed using the multiple linear stepwise regression method of the linear regression model. The final multiple linear regression model was statistically significant (F = 43.131, p < 0.001). A total of 50.7% of changes in the dependent variable can be explained by changes in independent variables (adjusted R2 = 0.507). The risk factors of fear of movement total score were older age, having local medical insurance, and lower education level. While the protective factors were having habits of exercising, higher levels of extraversion, conscientiousness, and emotional stability (**Figure 1A**; **Supplementary Table S5**).

## 4 Discussion

This cross-sectional study examined demographic and personality predictors of kinesiophobia in dialysis patients, as measured by the Tampa Scale for Kinesiophobia Heart (TSK-SV). The mean kinesiophobia score (45.128 ± 7.023) significantly exceeded the clinical threshold of 37 (49), indicating pronounced movement-related fear in this population - a novel finding in dialysis research.

Regression analyses identified advanced age as a significant predictor of elevated kinesiophobia scores across all subscales (fear,avoidance and dysfunction). This association may reflect age-related physiological declines, including: (1) progressive physical impairment (50), (2) compounded exercise limitations from renal comorbidities (51), and (3) heightened health vigilance characteristic of older adults (52). Together, these factors likely contribute to increased activity avoidance and movement apprehension in elderly dialysis patients.

Interestingly, being married was identified as a protective factor against the perception of danger. Married individuals had significantly lower danger scores compared to those with other marital statuses. Higher social support, which married people tend to have, suggests lower risk perception (53). This pattern might help account for the observed link between marital status and reduced fear of movement.

Regular exercise habits were also found to be a protective factor against fear of movement and its subscales. Studies have indicated that dialysis patients without long-term exercise habits are more susceptible to the side effects of dialysis, such as fatigue, hypotension, and muscle cramps. These side effects might be more noticeable during exercise, potentially contributing to higher fear of movement scores (54, 55).

The findings further suggest that certain personality traits significantly influence the fear of movement in dialysis patients. This underscores the need for personality assessments to study factors related to individual vulnerability and resilience in exercise interventions. A personalized psychological framework can enable individualized, progressive treatment plans, helping patients maximize their function and quality of life.

According to the Five Factor Model (56), Extraversion is characterized by assertiveness, sociability, and positive emotionality. Studies in chronic pain patients have found that Extraversion is negatively related to anxious and depressive symptoms, as well as fear-avoidance (57). Consistent with these findings, our study found that higher extraversion was associated with lower fear of movement. Patients with high Extraversion tend to report more optimism and positive attitudes toward their condition, which might correlate with greater adherence to medical advice and understand the positive effects of exercise.

In contrast, high levels of Neuroticism reveal a tendency to experience ambiguous emotions and interpret ordinary situations as threatening, viewing minor setbacks as significant difficulties (56). Previous research has shown a positive relationship between Neuroticism and fear-avoidance (57), supporting our findings that high Neuroticism is significantly associated with higher levels of fear of movement in dialysis patients. These patients tended to report more catastrophic interpretations of physical activity as a threat to kidney health, viewing rehabilitation as hopelessly difficult.

Conscientiousness reflects an individual’s tendency toward self-discipline, diligence, organization, and determination (56). Our results indicated that Conscientiousness was inversely associated with fear of movement. Highly conscientious individuals generally exhibit stronger goal-setting and planning behaviors. A study of peritoneal dialysis patients found that lower Conscientiousness was correlated with higher peritonitis risk (58). It is plausible that dialysis patients with higher Conscientiousness might demonstrate better adherence to collaboratively set health goals and establish structured routines, potentially contributing to more adaptive coping strategies.

Openness to experience encompasses traits such as curiosity, imagination, novelty-seeking, and cognitive flexibility (56). A cross-sectional study in CKD patients reported that Openness was associated with fewer health-related symptoms (59). Similarly, our study found that Openness was negatively associated with fear of movement. Patients with higher Openness scores tended to show greater willingness to consider new perspectives on exercise, which coincided with lower reported fear of movement.

The current results demonstrate significant associations between personality determinants and fear of movement in dialysis patients, suggesting their potential role as obstacles or enablers in multidisciplinary treatment programs. These findings emphasize the clinical value of incorporating personality assessments in pretreatment screening to identify patients with specific trait profiles who may require personalized rehabilitation approaches. While our cross-sectional design shows covariation rather than causation, the observed personality-kinesiophobia relationships tentatively parallel broader temperamental mechanisms described in affective disorders research (60), where non-pathological predispositions influence clinical presentations. This conceptual alignment raises the possibility that shared psychological features (e.g., threat monitoring tendencies and emotion regulation patterns) might underlie both domains, though future studies are needed to investigate whether temperament-informed interventions could be adapted for medically complex populations. The converging evidence highlights the necessity of further research examining personality traits in dialysis patients, particularly regarding their potential to modulate rehabilitation outcomes.

Despite several significant findings, this study has some limitations. The cross-sectional design precludes causal inferences and fails to capture temporal dynamics in the relationship between personality traits and kinesiophobia. Reliance on self-reported measures introduces potential social desirability and recall biases, particularly for sensitive items related to fear expression. Cultural influences on pain-related stoicism may further limit the generalizability of our findings across diverse populations. The absence of physiological markers (e.g., cortisol, heart rate variability) and a healthy control group restricts mechanistic interpretations and normative comparisons. Future research should employ longitudinal designs to establish temporal precedence, integrate multimodal assessments (behavioral tasks, biomarkers) to overcome self-report limitations, conduct cross-cultural validations to disentangle cultural moderators, and develop targeted interventions addressing personality-specific fear mechanisms. These advances would substantially strengthen both theoretical models and clinical applications.

## 5 Conclusions

Our study identified several significant predictors of kinesiophobia among dialysis patients. Multiple regression analyses revealed that advanced age, lower educational attainment, and lack of local medical insurance were independently associated with elevated kinesiophobia scores. Conversely, marital status and higher levels of extraversion, conscientiousness, openness, and emotional stability served as protective factors. We strongly recommend that nephrology teams incorporate psychological professionals to develop interdisciplinary rehabilitation plans tailored to dialysis patients’ specific personality profiles, particularly for those exhibiting elevated fear of movement.

## Data Availability

The original contributions presented in the study are included in the article/**Supplementary Material**. Further inquiries can be directed to the corresponding authors.
