# Supplementary material for: Personality traits: an important factor affecting fear of movement in dialysis patients
Source: Front Psychiatry. 2025 Jul 30;16:1574232. doi: 10.3389/fpsyt.2025.1574232 (PMC12343596; doi:10.3389/fpsyt.2025.1574232)
Supplement: Supplementary file 1 [file Table1.docx]

****Supplementary Material****

**Supplementary Table S1.** Multiple linear regression to predict Danger.

| **Variable** | **Standardized beta** | **B (95% CI)** | **P** |
| --- | --- | --- | --- |
| Marital status |  |  |  |
| Married |  |  | Ref |
| Other | 0.159 | 0.526 (0.201, 0.851) | 0.002** |
| Exercise |  |  |  |
| yes |  |  | Ref |
| no | 0.149 | 0.373 (0.117, 0.630) | 0.004** |
| Primary Disease |  |  |  |
| Diabetic Nephropathy | 0.124 | 0.386 (0.048, 0.724) | 0.026* |
| Hypertensive renal damage | 0.032 | 0.099 (-0.244, 0.443) | 0.569 |
| Chronic Nephritis | 0.158 | 0.461 (0.138, 0.784) | 0.005** |
| Other |  |  | Ref |
| Extraversion |  |  | NS |
| Agreeableness |  |  | NS |
| Conscientiousness |  |  | NS |
| Emotional Stability | -0.130 | -0.087 (-0.156, -0.018) | 0.013* |
| Openness | -0.270 | -0.117 (-0.163, -0.071) | <0.001*** |

Predictive variables tested by Stepwise method.

*p < 0.05, **p <0.01, ***p < 0.001.

**Supplementary Table S2.** Multiple linear regression to predict Fear.

| **Variable** | **Standardized beta** | **B (95% CI)** | **P** |
| --- | --- | --- | --- |
| Age | 0.314 | 0.046 (0.032, 0.060) | <0.001*** |
| Marital status |  |  | NS |
| Married |  |  |  |
| Other |  |  |  |
| Medical insurance |  |  |  |
| Local |  |  | Ref |
| Not local | -0.125 | -1.025 (-1.755, -0.296) | 0.006** |
| Education |  |  |  |
| Junior middle school or below | 0.167 | 0.736 (0.098, 1.375) | 0.024* |
| High school or junior college | 0.228 | 1.001 (0.379, 1.623) | 0.002** |
| Bachelor degree or above |  |  | Ref |
| Alcohol and tobacco use |  |  |  |
| yes |  |  | Ref |
| no | 0.094 | 0.732 (0.029, 1.435) | 0.041* |
| Exercise |  |  |  |
| yes |  |  |  |
| no | 0.291 | 1.275 (0.856, 1.694) | <0.001*** |
| Initial dialysis modality |  |  | NS |
| Hemodialysis(HD) |  |  |  |
| Peritoneal dialysis (PD) |  |  |  |
| Current dialysis modality |  |  | NS |
| HD |  |  |  |
| PD/HD+PD |  |  |  |
| Conscientiousness | -0.142 | -1.048 (-0.246, -0.051) | 0.003** |
| Openness |  |  | NS |

Predictive variables tested by Stepwise method.

*p < 0.05, **p <0.01, ***p < 0.001.

**Supplementary Table S3.** Multiple linear regression to predict Avoidance.

| **Variable** | **Standardized beta** | **B (95% CI)** | **P** |
| --- | --- | --- | --- |
| Age | 0.325 | 0.070 (0.051, 0.089) | <0.001*** |
| Medical insurance |  |  |  |
| Local |  |  | Ref |
| Not local | -0.140 | -1.696 (-2.691, -0.701) | 0.001** |
| Education |  |  |  |
| Junior middle school or below | 0.167 | 1.081 (0.213, 1.948) | 0.015* |
| High school or junior college | 0.184 | 1.184 (0.336, 2.031) | 0.006** |
| Bachelor degree or above |  |  | Ref |
| Alcohol and tobacco use |  |  | NS |
| yes |  |  |  |
| no |  |  |  |
| Exercise |  |  |  |
| yes |  |  | Ref |
| no | 0.340 | 2.189 (1.615, 2.762) | <0.001*** |
| Initial dialysis modality |  |  | NS |
| Hemodialysis(HD) |  |  |  |
| Peritoneal dialysis (PD) |  |  |  |
| Current dialysis modality |  |  | NS |
| HD |  |  |  |
| PD/HD+PD |  |  |  |
| Extraversion | -0.120 | -0.123 (-0.210, -0.036) | 0.006** |
| Agreeableness |  |  | NS |
| Conscientiousness | -0.169 | -0.260 (-0.393, -0.126) | <0.001*** |
| Emotional Stability |  |  | NS |
| Openness |  |  | NS |

Predictive variables tested by Stepwise method.

*p < 0.05, **p <0.01, ***p < 0.001.

**Supplementary Table S4.** Multiple linear regression to predict Dysfunction.

| **Variable** | **Standardized beta** | **B (95% CI)** | **P** |
| --- | --- | --- | --- |
| Age | 0.203 | 0.030 (0.017, 0.044) | <0.001*** |
| Exercise |  |  |  |
| yes |  |  | Ref |
| no | 0.284 | 1.259 (0.848, 1.670) | <0.001*** |
| Primary Disease |  |  |  |
| Diabetic Nephropathy | 0.095 | 0.523 (0.007, 1.039) | 0.047* |
| Hypertensive renal damage | 0.080 | 0.441 (-0.082, 0.963) | 0.098 |
| Chronic Nephritis | 0.120 | 0.618 (0.128, 1.108) | 0.014* |
| Other |  |  | Ref |
| Extraversion | -0.162 | -0.114 (-0.187, -0.042) | 0.002** |
| Agreeableness |  |  | NS |
| Conscientiousness | -0.123 | -0.131 (-0.230, -0.032) | 0.010* |
| Emotional Stability | -0.128 | -0.152 (-0.260, -0.044) | 0.006** |
| Openness | -0.145 | -0.111 (-0.198, -0.025) | 0.012* |

Predictive variables tested by Stepwise method.

*p < 0.05, **p <0.01, ***p < 0.001.

**Supplementary Table S5.** Multiple linear regression to predict Fear of movement total score.

| **Variable** | **Standardized beta** | **B (95% CI)** | **P** |
| --- | --- | --- | --- |
| Age | 0.311 | 0.147 (0.107, 0.187) | <0.001*** |
| Medical insurance |  |  |  |
| Local |  |  | Ref |
| Not local | -0.100 | -2.652 (-4.700, -0.604) | 0.011* |
| Education |  |  |  |
| Junior middle school or below | 0.161 | 2.279 (0.477, 4.082) | 0.013* |
| High school or junior college | 0.186 | 2.625 (0.107, 0.187) | 0.003** |
| Bachelor degree or above |  |  | Ref |
| Exercise |  |  |  |
| yes |  |  | Ref |
| no | 0.374 | 5.264 (4.068, 6.459) | <0.001*** |
| Initial dialysis modality |  |  | NS |
| Hemodialysis(HD) |  |  |  |
| Peritoneal Dialysis (PD) |  |  |  |
| Current dialysis modality |  |  | NS |
| HD |  |  |  |
| PD/HD+PD |  |  |  |
| Extraversion | -0.144 | -0.323 (-0.505, -0.141) | 0.001** |
| Agreeableness |  |  | NS |
| Conscientiousness | -0.183 | -0.618 (-0.897, -0.339) | <0.001*** |
| Emotional Stability | -0.102 | -0.384 (-0.697, -0.071) | 0.016* |
| Openness |  |  | NS |

Predictive variables tested by Stepwise method.

*p < 0.05, **p <0.01, ***p < 0.001.
